# Annual acknowledgement of reviewers

**DOI:** 10.1186/1472-698X-13-14

**Published:** 2013-03-19

**Authors:** Emily Crow

**Affiliations:** 1 BioMed Central 236 Grays Inn Rd, London, WC1X 8HB, United Kingdom

## Abstract

**Contributing reviewers:**

The editors of *BMC International Health and Human Rights* would like to thank all our reviewers who have contributed to the journal in Volume 12 (2012).

## 

Aniekan Abasiattai

Nigeria

Nadia Abdulhadi

Sweden

Adebola Adedimeji

USA

Ramesh Adhikari

Nepal

Suneth Agampodi

Sri Lanka

Irene Akua Agyepong

Ghana

Syed Masud Ahmed

Bangladesh

Jennifer Ailshire

USA

Arshad Altaf

Pakistan

Aranka Anema

Canada

Uzochukwu Aniebue

Nigeria

Ludwig Apers

Belgium

Shams Arifeen

Bangladesh

Amir Attaran

Canada

Miroslav Backonja

USA

Marian Barnes

United Kingdom

Mickael Bech

Denmark

Susanne Bejerot

Sweden

Tefera Belachew

Ethiopia

Katrien Benhalima

Belgium

Corina Benjet

Mexico

Yemane Berhane

Ethiopia

Xavier Bosch-Capblanch

Switzerland

Marek Brabec

Czech Republic

Malcolm Bryant

USA

Luis Cabero

Spain

J. Gary Cameron

Canada

Tim Carey

USA

Gonca Celik

Turkey

Likwang Chen

Taiwan

Matthew Chersich

South Africa

Bright Chigbu

Nigeria

Malcolm Clarke

United Kingdom

John Cleland

United Kingdom

Bart Criel

Belgium

Wendy Cross

Australia

Koustuv Dalal

Sweden

Yoswa Dambisya

South Africa

Nihaya Daoud

Israel

Manuela De Allegri

Germany

Ayesha De Costa

Sweden

Ilse Derluyn

Belgium

Mamuka Djibuti

Georgia

Mai Do

USA

Lena Dohlman

USA

Hengjin Dong

China

Martin Dunser

Austria

Karen Eggleston

USA

Maria Ekstrand

USA

Ariel Eytan

Switzerland

Thomas Faunce

Australia

Jane Ferguson

Switzerland

Elisabeth Fichet-Calvet

Germany

Sharon Fonn

South Africa

Tanja Franciskovic

Croatia

Paul Freeman

USA

Jacqueline Gahagan

Canada

Natalia Gavrilova

USA

Robert Geneau

Canada

Lan Gien

Canada

Lucy Gilson

South Africa

Nancy Glass

USA

John Grundy

Cambodia

Oye Gureje

Nigeria

Brian Gushulak

Canada

Adrian Guta

Canada

Tariq Hasoon

Iraq

Katarina Hjelm

Sweden

Katherine Homewood

United Kingdom

Kun Huang

China

Vincent Iacopino

USA

David Jacobs

USA

Sara Javanparast

Australia

Caroline Jehu-Appiah

Tunisia

Linda Kahn

USA

Niranjan Karnik

USA

Getnet Kassie

Ethiopia

Rebecca Katz

USA

Theodoros Kelesidis

USA

Ali Khaji

Iran

Mahmud Khan

USA

Kazuko Kimura

Japan

Kairi Kolves

Australia

Elena Krumova

Germany

Anil Kumar

India

Vishwajeet Kumar

India

Charles Larson

Canada

Jennifer Leaning

USA

Christos Lionis

Greece

Kawika Liu

USA

Scott Lorch

USA

John Maluccio

USA

Alexandra Martiniuk

Australia

Catherine Mathews

South Africa

Joseph Matovu

Uganda

Godfrey Michael Mbaruku

Tanzania

Girmay Medhin

Ethiopia

Benjamin Meier

USA

Sharmila Mhatre

Canada

Steven Mitchell

Canada

Anietie Moses

Nigeria

Malgorzata Mossakowska

Poland

Zubia Mumtaz

Canada

Bavon Mupenda

Congo Democratic Republic

Valbona Muzaka

United Kingdom

Noeline Nakasujja

Sweden

Eileen Natuzzi

USA

Mzikazi Nduna

South Africa

Peter A Newman

Canada

Olumuyiwa Odusanya

Nigeria

Bolajoko Olusanya

Nigeria

Sunday Omilabu

Nigeria

Gorik Ooms

Belgium

Joanna Orne-Gliemann

France

Kayhan Parsi

USA

Mahomed Patel

Australia

Maria Lucia Penna

Brazil

Inge Petersen

South Africa

Nittaya Phanuphak

Thailand

Anastas Philalithis

Greece

Emanuele Pontali

Italy

Helen Potts

USA

Barbara Preitler

Austria

Gisela Priebe

Sweden

Guitele Rahill

USA

Mosiur Rahman

Japan

Fahimeh Ramezani Tehrani

Iran

Vibeke Rasch

Denmark

Mohammad-Reza Rasouli

Iran

Naghma Rehan

Pakistan

Michael Reich

USA

Melvin Reynolds

United Kingdom

Haris Riaz

Pakistan

Jacquie Ripat

Canada

James Ron

USA

Michael Rowe

USA

Eduardo Salazar-Lindo

Peru

Miguel San Sebastian

Sweden

Ingvild Sandøy

Norway

Leonor Maria Pacheco Santos

Brazil

Catherine Sauvaget

France

Enid Schatz

USA

Jean Schensul

USA

Ted Schrecker

Canada

Janet Seeley

Uganda

Charles Senessie

Switzerland

Asrul Shafie

Malaysia

Babar Tasneem Shaikh

Pakistan

Leickness Chisamu Simbayi

South Africa

Kenneth Simiyu

Canada

Melissa Simon

USA

Seter Siziya

Zambia

Jeremy Snyder

Canada

Steven Stack

USA

Rob Stephenson

USA

Maria Stuttaford

Netherlands

Charlotte Tawiah-Agyemang

Ghana

Sharon Thompson

USA

Keith Tin

Hong Kong

Jurrien Toonen

Ghana

Kwasi Torpey

Ghana

Andreas Valentin

Austria

Ravi Verma

India

Maretha Visser

South Africa

Mark Wahlqvist

Taiwan

Joyce Wamoyi

Tanzania

Shr-Jie Wang

Denmark

Thomas Wenzel

Austria

Albert Wertheimer

USA

Ben White

USA

Amanda Williams

United Kingdom

Daniel Fekadu Wolde-Giorgis

United Kingdom

Yao-Jong Yang

Taiwan

Eyob Zere

Zimbabwe

## Pre-publication history

The pre-publication history for this paper can be accessed here:

http://www.biomedcentral.com/1472-698X/13/14/prepub

